# Traumatic hepatic arteriohepatic venous fistula managed with selective coil embolization: a case report

**DOI:** 10.1259/bjrcr.20150512

**Published:** 2017-01-07

**Authors:** Rajsekar Chandrasekharan, Sreekumar KP, Srikanth Moorthy, Chinmay Kulkarni

**Affiliations:** Amrita Institute of Medical Sciences and Research Center, Kochin, India

## Abstract

Hepatic arterioportal fistulae are frequent vascular complications due to neoplasm, trauma and iatrogenic injury. On the other hand, fistulae between the hepatic arteries and hepatic veins (arteriohepatic venous fistula) are rare. We report the case of a 45-year-old male who suffered from a blunt abdominal trauma with abdominal distension. Initial cross-sectional imaging revealed laceration of the right lobe of liver with an arteriovenous fistula and hemoperitoneum. The diagnosis of arteriohepatic venous fistulae was confirmed on digital subtraction angiography (DSA) and treated angiographically with superselective coil embolization. Post-embolization angiogram showed complete occlusion of arteriovenous fistulae. We emphasis on the management part of the fistulae and endovascular treatment.

## Background

Hepatic arterioportal fistulae are frequent vascular complications due to neoplasm, trauma and iatrogenic injury. On the other hand, fistulae between the hepatic arteries and hepatic veins (arteriohepatic venous fistula) are rare. We report the case of a 45-year-old male who suffered from a blunt abdominal trauma with abdominal distension. Initial cross-sectional imaging revealed laceration of the right lobe of liver with an arteriovenous fistula and hemoperitoneum. The diagnosis of arteriohepatic venous fistulae was confirmed on digital substraction angiography (DSA) and treated angiographically with superselective coil embolization. Post-embolization angiogram showed complete occlusion of arteriovenous fistulae. Hung et al^1^ has previously reported a similar case with the same diagnosis using multidimensional computed tomography (MDCT) angiography. We emphasis on the management part of the fistulae and endovascular treatment.

## Case report

A 45-year-old male with history of blunt abdominal trauma was brought to casualty with abdominal distension and drop in haemoglobin levels. Initial ultrasound imaging revealed free fluid in the abdomen. Exploratory laprotomy was done that show hemoperitoneum, and liver laceration in the right lobe. Perihepatic packing was done to achieve hemostasis.

**Figure 1.**
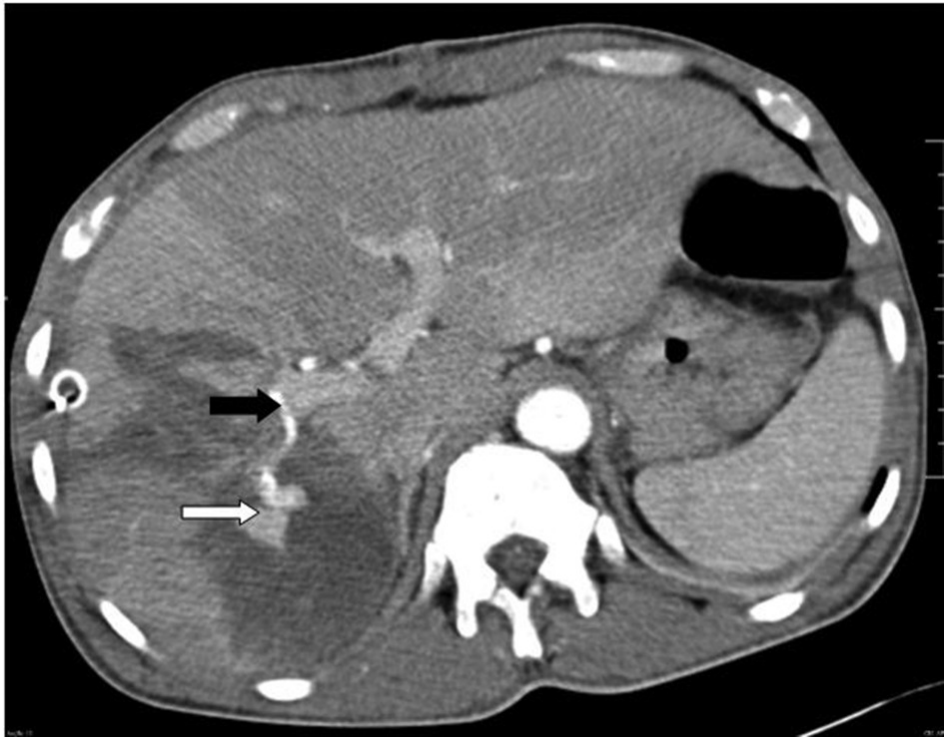
A 50-year-old male with history of blunt trauma abdomen. MDCT arterial phase image shows right hepatic artery (black arrow) distally showing ectatic arterial lumen (white arrow). The area of hypo attenuation adjacent to the ectatic vessel represents hepatic parenchymal injury.

**Figure 2.**
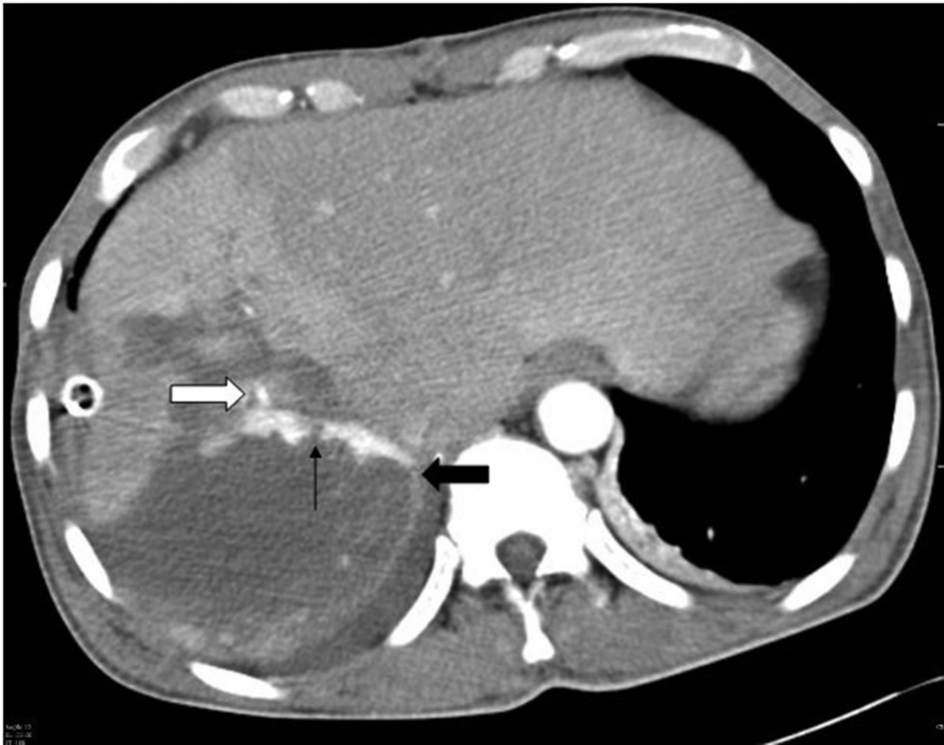
MDCT arterial phase image shows the segmental branch (white arrow) of right hepatic artery communicating with the linear hyperdense fistulous track (thin black arrow) draining towards the right hepatic vein (thick black arrow).

CT imaging done on day two revealed replaced right hepatic artery arising from superior mesenteric artery (SMA) with hepatic laceration involving segment VI and VII. Arterial phase CT images showed a prominent segmental branch of the right hepatic artery with a fistulous track communicating with the right hepatic vein (Figures 1–2). Two days later in view of the further drop in hemoglobin and rising SGPT/SGOT levels, it was decided to intervene.

**Figure 3.**
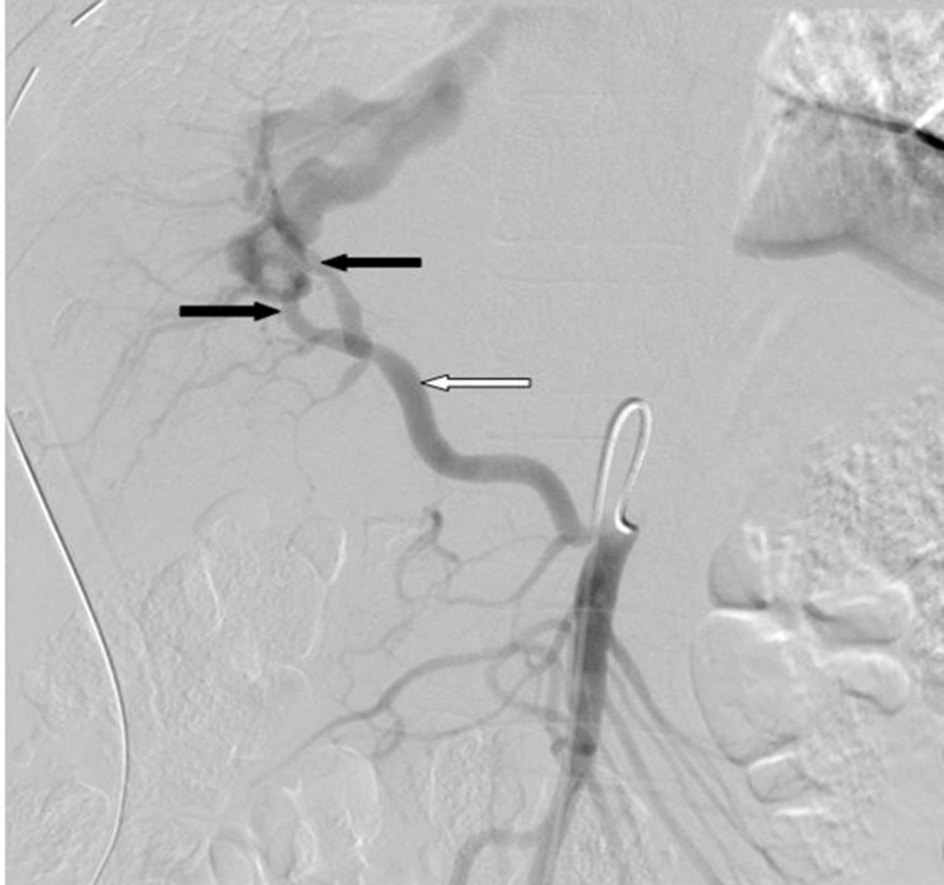
Angiographic images show replaced right hepatic artery originating from the SMA (white arrow). The segmental branches arising from the right hepatic artery communicate with the fistulous tracks (black arrows) draining into the hepatic vein.

**Figure 4.**
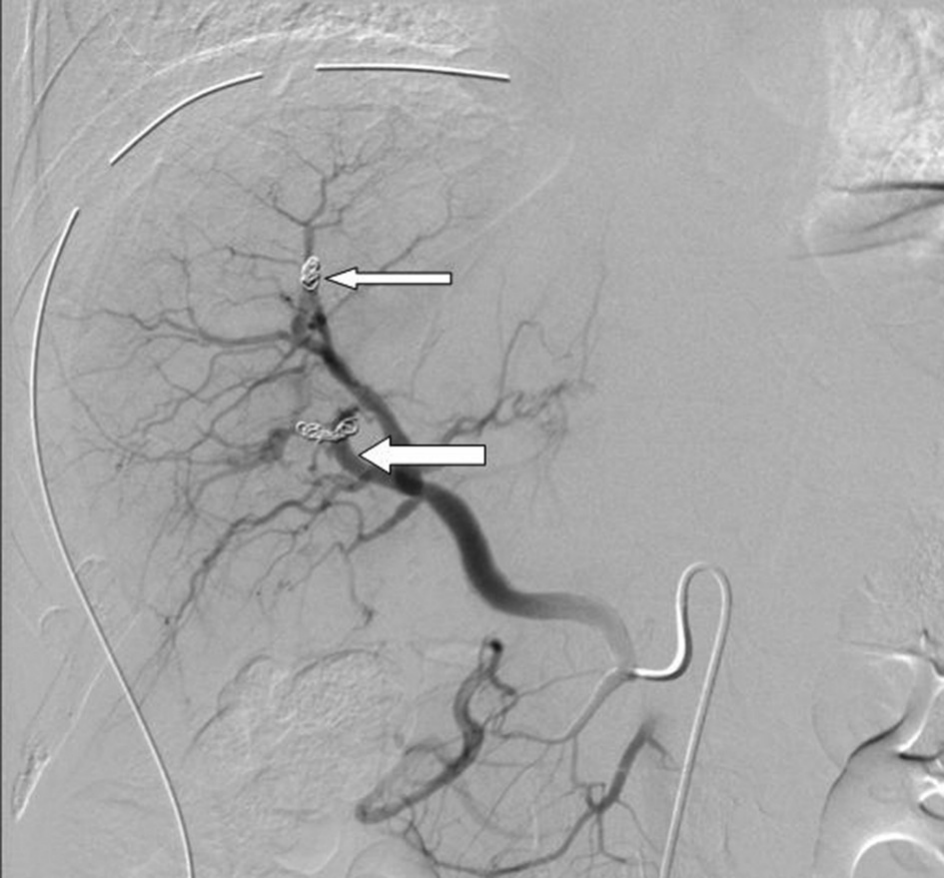
Status post-deployment of coils in both the fistulous tracks. Angiogram shows (white arrows) completely occluded AV fistulous tracks with good arborization of hepatic arterial branches within the hepatic parenchyma.

From a transfemoral approach, the superior mesenteric artery was catheterized with 6F catheter (Chuang-William Cook Europe APS). Selective angiogram showed two prominent hepatic artery segmental branches leading to the lacerated liver parenchyma with early filling of the right hepatic vein suggesting post-traumatic arteriovenous fistulae (Figure 3). The replaced right hepatic artery was catheterized coaxially with a progreat microcatheter (Terumo Corporation, Tokyo, Japan) and then navigated into the segmental arterial branches leading to the arteriovenous fistulae. The segmental branches leading to the arteriovenous fistulae were embolized with multiple 018 micro coils (Tornado Embolisation Coils Cook, Bloomington, IL). Post-embolization angiography showed completely occluded arteriovenous fistulae (Figure 4). The patient was followed up for 2 weeks and he made an uneventful recovery.

## Discussion

The most common cause of liver injury is blunt abdominal trauma. The incidence of hepatic vascular injuries after blunt trauma is much lower than that after penetrating injury. Traumatic intrahepatic arteriovenous fistulae are rarely encountered which generally involve arterial to portal venous connection (arterioportal shunts) and rarely result in arterial to hepatic vein connection. The leading cause of arteriovenous fistulae are neoplasm,^2^ congenital anomaly,^3^ trauma and iatrogenic injuries like needle biopsy and percutaneous drainage.^4^

Okuda et al^4^ in his series of patients after needle biopsy and percutaneous biliary drainage demonstrated arterioportal fistula in eight patients and arteriohepatic venous fistula in one patient on angiography. Fistulae of systemic venous system can lead to cardiovascular compromise with high output cardiac failure.^5^ Infrequent cause of arteriohepatic venous fistulas may be attributed to the larger distance between artery and hepatic vein. Post-traumatic arterioportal shunts are more frequent as these vessel systems are in close proximity at the portal tract. Dessousky et al^6^ in his series of patients with intrahepatic vascular shunts proposed a strategy for early identification, classification and management of shunts.

Intrahepatic vascular shunts (IHVSs) are abnormal communications between intrahepatic vasculature involving the arterial, portal or hepatic venous systems. The etiology of these shunts is controversial and may be either acquired, as those associated with cirrhosis or hepatocellular carcinoma, those that occur after traumatic injuries to the liver or interventional transhepatic procedures (including liver biopsy, transhepatic cholangiography or biliary surgery), or they may appear in the form of congenital and idiopathic vascular malformations, as in Rendu–Osler–Weber syndrome (hereditary hemorrhagic telangiectasia).^7^ Congestive cardiac failure, portal hypertension, portosystemic encephalopathy, cholangitis and atypical cirrhosis have been reported as possible serious complications related to this condition. Thus, a correct diagnosis is important and diagnostic imaging has a fundamental role in the evaluation of shunts and determination of the appropriate management.^8^ Two types of shunts were identified- arteriovenous (72%) and venovenous (28%). Arteriovenous shunts can be further classified into arterioportal and arteriohepatic shunts, whereas venovenous shunts can be further classified into portovenous, portoportal and venovenous shunts.^6^

Dessousky et al^6^ proposed a practical strategy with therapeutic implication based on both imaging and clinical findings and categorized patients into three groups as follows: Group I included asymptomatic patients with small non-neoplastic shunts (3–6 mm) and with shunt ratio < 30%. They were followed by Doppler and clinical examination at 3 to 6 months interval. If the shunt did not change or regressed, and no signs or symptoms developed, then conservative management was applied according to the type of shunt.

Group II included patients with symptomatic shunts, large (15–23 mm) or aneurysmal shunt (28–45 mm), those with shunt ratio >30% or neoplastic shunt. These shunts are usually managed by interventional technique.

Group III included patents with diffuse shunts or with shunt ratio >60%, and were planned directly for surgery.

In our case, as the patient was symptomatic and as the size of the fistulae were >15 mm, management with embolization was considered.

3D post-processing of CT angiography with multiplanar reformation (MPR), maximal intensity projection (MIP) and volume rendering technique (VRT) is especially helpful in detection of vascular injuries. In this case, CT promptly showed the hypertrophic segmental branches leading to the fistulae. Interventional techniques, especially angiographic embolization, are well-known as useful adjunct in non-operative management of blunt liver trauma.^9^ Early angiographic embolization decreases the incidence of blood transfusion and number of liver-related operations.^8^ Transcatheter arterial embolization has proved to be effective in treatment of arterioportal shunts, and its associated complications are rare.^10^

## Conclusions

Arteriovenous shunts are rare complication of blunt trauma. Arteriohepatic shunts are rarer than arterioportal shunts. Multiphasic MDCT can be very helpful in proper planning before an interventional procedure. Angiographic embolization can decrease the incidence of transfusion and surgical procedures. Intrahepatic vascular shunts can be managed by interventional technique, and surgery depending on clinical presentation and shunt ratio.

## Learning points

1. Arteriohepatic venous shunts can occur rarely when compared with arterioportal shunts in abdominal trauma.
2. Management of arteriohepatic venous shunts is similar to arterioportal shunts by interventional technique like micro coil embolization depending on the shunt ratio and clinical presentation.

## Consent

Written informed consent was obtained from the patient(s) for publication of this case report, including accompanying images.
